# Differential genotype response to increased resource abundance helps explain parallel evolution of *Daphnia* populations in the wild

**DOI:** 10.1002/ece3.9896

**Published:** 2023-03-15

**Authors:** Kelsey Lyberger, Thomas W. Schoener

**Affiliations:** ^1^ Department of Evolution and Ecology, Center for Population Biology University of California Davis Davis California USA

**Keywords:** *Daphnia*, natural selection, parallel evolution, trade‐off

## Abstract

Under controlled laboratory conditions, previous studies have shown that selection can produce repeatable evolutionary trajectories. Yet, the question remains for many of these studies if, given identical starting populations, evolution in the wild proceeds in a non‐random direction. In the present study, we investigated the extent to which rapid evolution in the wild is parallel by monitoring the genetic composition of replicate populations of *Daphnia* in field mesocosms containing two clonal genotypes. We found parallel changes across all nine mesocosms, in which the same genotype increased in frequency. To probe whether genotype‐specific response to resource abundance could have led to this frequency change, we conducted a life‐history assay under high‐resource abundance and low‐resource abundance. We found that resource exploitation differed by genotype, in that, while one genotype (the winner in the field mesocosms) was more fit than the other genotype at high resources, the other genotype performed slightly better at low resources. We suspect that levels of resource abundance found in the summer field mesocosms had values in which the genotype better with abundant resources had the advantage. These findings suggest that variation in certain traits associated with resource acquisition can drive genotype frequency change.

## Graphical abstract

In this study, we test the extent to which rapid evolution in the wild is repeatable and whether genotype‐specific sensitivity to resource abundance may be an important selective mechanism that shapes this evolutionary trajectory. We find parallel changes across all mesocosms containing *Daphnia*, in which the same genotype increased in frequency. Also, we find that resource exploitation differed by genotype which indicates a potential resource‐based trade‐off.

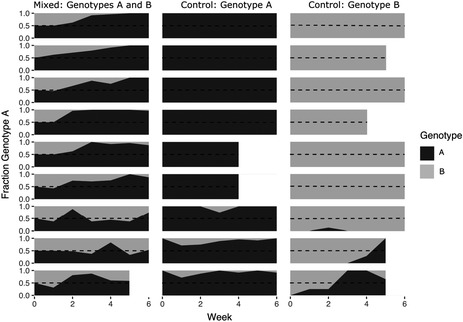

## 1 INTRODUCTION

A growing body of work in experimental evolution, spurred by Stephen Jay Gould's idea of “replaying life's tape”, has begun to evaluate whether evolution follows a deterministic route in response to some environmental pressure (reviewed in Blount et al., 2018; Lobkovsky & Koonin, 2012; Losos, 2017). The question remains: does the deterministic force of selection produce repeatable evolutionary trajectories or does chance dominate? Previous experiments, typically limited to microbes in laboratory settings and highly controlled environments, have shown a relatively high degree of repeatability in evolutionary responses (e.g., Bull et al., 1997; Lenski et al., 1991). Some field studies, for example, in Trinidadian guppies and three‐spine sticklebacks, have demonstrated parallel trait change in response to environmental pressures, but there is high variation in the extent of parallelism among studies (Kaeuffer et al., 2012; Oke et al., 2017; Reznick & Endler, 1982). Furthermore, in using sexual species, such studies are limited in their ability to create identical starting populations on which selection can act. In this study, we are interested in understanding the extent to which rapid evolution in the wild is predictable and whether genotype‐specific sensitivity to resource abundance may be an important selective mechanism that shapes this evolutionary trajectory.

The freshwater crustacean, *Daphnia pulex*, provides an excellent model system in which to address these questions because of their short generation times and cyclically parthenogenetic mode of reproduction. Under favorable conditions, females reproduce clonally, then switch to sexual reproduction as conditions become less favorable, typically during winter months. Because of its clonal nature, we are able to rear multiple genetically identical populations on which selection can act. Additionally, *Daphnia* populations are easily maintained in field mesocosm experiments in which they are exposed to ambient temperature and light, as well as to natural phytoplankton communities. These advantages make conducting a “parallel replay experiment” in the wild feasible. While previous observational studies have documented genotype turnover in the wild (e.g., Steiner & Nowicki, 2019) and demonstrated similarity across multiple locations within a lake and across years (Carvalho & Crisp, 1987), to our knowledge, there has yet to be an experimental study documenting parallel genotype frequency changes across replicated populations in the wild.

One of the most likely drivers of a deterministic outcome in *Daphnia* is resource availability, and previous laboratory microcosm experiments in *Daphnia* have demonstrated that algal resource conditions can drive evolution (Drugă et al., 2016; Weider et al., 2005). Resource availability is a constraining feature of many environments, under which differences in a genotype's ability to exploit resources will be directly reflected in its fitness. However, a high‐exploitation strategy may not always be favored, in that performing especially well in a high‐resource environment may trade‐off with performing especially poorly in a low‐resource environment (Reznick et al., 2000). In resource‐poor environments, a less reactive or K‐selected strategy, for example, efficient metabolic processing, that performs better under low‐resource conditions would be favored because their metabolisms require fewer resources for growth and reproduction.

Here, we present the results of two interrelated experiments. First, to understand to what extent genotype frequency change is predictable, we monitored the genetic composition of replicate populations in field mesocosms initiated with an equal proportion of two genotypes. With strong selection we expect frequencies to change in the same direction to favor one genotype over the other in all populations; whereas, without selection, we expect frequencies to drift randomly. Second, to understand whether resource abundance is a potential driver of this frequency change, we conducted a life‐history assay to assess whether the genotypes exhibit fitness differences under high‐ and low‐resource conditions.

## 2 METHODS

### 2.1 Sample collection and rearing

In May 2018, we sampled *D. pulex* clones from a mesotrophic pond at UC McLaughlin Reserve, CA (38.870417, −122.428917). We took a vertical plankton tow from the center and deepest part of the pond, roughly 30 m from shore. Located in a Mediterranean climate, this pond remains habitable for *Daphnia* year‐round. The pond has a maximum depth of 6 m and a surface area of 3600 m^2^. During our initial sample, the chlorophyll a content was 3.0 μg L^−1^ and the mean across all 6 weeks of the experiment was 4.9 μg L^−1^ (SD = 1.4). During the experiment, we recorded the composition of major zooplankton taxa in the pond and the temperature of the pond at the surface and 3 m depth (Table A1 in the Appendix). *D. pulex* was the only *Daphnia* species observed, although *Daphnia dentifera* have been observed in other years. We genotyped a random sample of 16 isolates from the initial sample at five microsatellite markers (see Latta et al., 2010 for a detailed description of these markers). We found the population was dominated by two multilocus genotypes (Table 1). The probability of two individuals having identical genotypes at all five loci was <.001 (Table A2 in the Appendix; Taberlet & Luikart, 1999). The two dominant genotypes—hereafter called genotypes A and B—can be distinguished from each other at a single locus (CAA2) using gel electrophoresis (either a single band or two bands). This method allowed for the genotyping of thousands of individuals (*N* = 2874). Starting from a single gravid female, we clonally propagated thousands of *Daphnia* of genotype A. We did the same for genotype B. These batch cultures were used to initiate our mesocosm experiment and to run life‐history assays. Given there were no males or resting eggs in the batch cultures, we can assume that all individuals stayed genetically identical.

**TABLE 1:** Multilocus genotypes of the two dominant genotypes A and B. The two genotypes differ at all five microsatellite markers. Gel electrophoresis run on marker “CAA2” allowed us to distinguish between the two genotypes. Primers for these markers are from Colbourne et al. (2004).

	CAA8		GTT3		CAA27		CAA2		CAA14	
Genotype A	136	148	202		199	205	213	224	243	246
Genotype B	130	142	198	202	203	206	223	224	240	243

### 2.2 Experiment 1: Determining natural changes in gene frequency

In June 2018, we set out a total of 27 floating mesocosms, divided into three treatments: an equal mix of the two genotypes, and each genotype alone. The former allowed us to determine whether selection was acting to favor one genotype over the other or whether genotype frequency change was driven by chance. The latter were used as controls which allowed us to determine whether populations of each genotype alone were able to grow under these semi‐natural conditions. The mesocosms were placed approximately 15 m from shore (38.87057, −122.42902) and floated on the surface of the pond, anchored by weights.

To create the mesocosms, we floated clear LDPE plastic bags in the pond and filled each with 50 L of pond water that had been filtered through a 63 μm mesh to remove all zooplankton. The dimensions of a mesocosm were approximately 0.53 m deep by 0.42 m wide. We covered them with mesh lids to prevent falling leaves or other detritus from entering. We waited 5 days then added 39 individual *Daphnia* of genotype A and 39 of genotype B to each of nine mesocosms. As a single genotype control, we added 78 *Daphnia* of genotype A to a second set of nine mesocosms and 78 *Daphnia* of genotype B to a third and final set of nine mesocosms. The initial age structure for all mesocosms started as 30 juveniles, 16 gravid females, and 32 non‐gravid females. All starting densities were approximately 1.56 L^−1^.

We sampled all mesocosms every week for 6 weeks, during which a maximum of approximately six generations occurred, given that the average age at maturity is 7 days. To measure density, we sampled 9‐L columns of water with a 13‐cm‐diameter, 153‐μm‐mesh zooplankton net towed upward from the bottom of the mesocosm, repeated 2–6 times. To characterize relative changes in chlorophyll a, we used the Total Algae PC Smart Sensor on a Yellow Springs Instrument EXO2 water quality sonde. To measure genotype frequency change, we genotyped 24 individuals, or all individuals collected if <24, for mixed genotype mesocosms and 8 individuals for those with only one genotype. Six of 18 single‐genotype mesocosms became contaminated with a second genotype during the course of the experiment. In these exceptional cases, we genotyped up to 24 individuals after identifying that contamination had occurred (i.e., starting week 4 because there was a 3‐week delay in the time it took to perform the genotyping). We ended the experiment after 6 weeks, by which point populations in six mesocosms had declined to extinction and one mesocosm had been punctured by a fishing hook.

### 2.3 Experiment 2: Life‐history assay

In a controlled laboratory setting, we crossed two genotypes with two levels of resources. We measured individual life histories of genotype A and genotype B under high (16.5 μg L^−1^ chlorophyll a)‐ and low (0.91 μg L^−1^ chlorophyll a)‐resource conditions. We measured three traits: individual growth rate, age at maturity, and reproductive output. The resource conditions were chosen based on a previous study in *Daphnia* showing growth rates saturate at 15 μg L^−1^ when fed with natural phytoplankton (Müller‐Solger et al., 2002). Because our life‐history assay used lab‐grown monocultures of phytoplankton, which are typically higher‐quality food than natural phytoplankton, the high concentration used is likely to be beyond the saturation point. For similar reasons, the resource availability in the life‐history assay and the field mesocosms cannot be directly compared. The laboratory monoculture will have different chlorophyll a fluorescence, edibility, and nutritional value than the natural phytoplankton community.

We conducted the life‐history assay on 10 individuals per genotype and treatment (*N* = 40). However, three individuals died before their individual growth rate could be measured. For genotype A, sample sizes were 8 high and 10 low, and for genotype B, sample sizes were 10 high and 9 low. Prior to the start of the assay, to eliminate maternal and grandmaternal effects, one adult from each batch culture was selected and propagated for three generations using the second clutch. To begin the assay, neonates <12 h old were then selected to be measured. All *Daphnia* were reared in individual containers filled with 200 mL filtered pond water (mesh size of 0.7 μm) and kept under controlled laboratory conditions (16 L:8D, at 20°C). Every other day we changed the water in each container and fed cultures 400 μL of *Nannochloropsis* sp. in the high‐resource condition (1.2 × 10^8^ cells mL^−1^) and 40 μL in the low‐resource condition (1.2 × 10^7^ cells mL^−1^) to maintain approximately constant chlorophyll a concentrations over time. Every day the location of the containers in the incubation chamber was randomized.

We recorded individual growth rate of juveniles, age at maturity, and reproduction. To measure growth rate, we photographed individuals on day 1 and day 4 with a Cannon EOS Rebel T3i camera mounted to a microscope at 20× magnification to produce a 3400 × 5100 pixel jpeg image. Size was measured manually using ImageJ by drawing a line segment from the base of the tail to top of the eye. By measuring a single image repeatedly, we found this method produces a measurement error of SD = 0.009 mm. We then used these two sizes to estimate growth rate for each individual in mm day^−1^. One photograph from day 1 (genotype B/low) was missing, so a growth rate could not be determined for that individual. To obtain an estimate of the age at maturity, we monitored individuals for the presence of eggs every 12 h. Finally, to measure reproductive output, we summed the number of neonates produced in an individual's first three clutches. Because the interval between clutches is longer than the interval between changing out the water and container, we were able to count a clutch of neonates and then move the adult female into her next container.

### 2.4 Data analysis

All statistics were performed using R version 3.6 (R Development Core Team, 2021). A Wilcox signed‐rank test evaluated whether final genotypic frequency of genotype A differed significantly from a frequency of 0.5 across all two‐genotype mesocosms (*n* = 9), a result that would indicate selection (i.e., a deterministic outcome). In contrast, if the frequency did not differ significantly from 0.5, this would provide evidence that changes to genotypic frequencies were stochastic.

For life‐history assays, we evaluated the effects of genotype, resource condition, and their interaction on the measured life‐history traits using a MANOVA test. To determine the effects of genotype, resource condition, and their interaction on specific traits, we followed the MANOVA with three separate two‐way ANOVAs, one for each of the primary response variables: growth, age at maturity, and reproduction. To meet the assumption of normality, data for age at maturity and reproduction were natural log transformed. While we expected to see an effect of resource condition alone on our measures of fitness, an interaction between genotype and resources would indicate a resource‐based trade‐off between the two genotypes. We followed this analysis with pairwise comparisons of the genotypes within each resource treatment and reported Bonferroni‐adjusted *p*‐values using the package “emmeans.”

## 3 RESULTS

**FIGURE 1:**
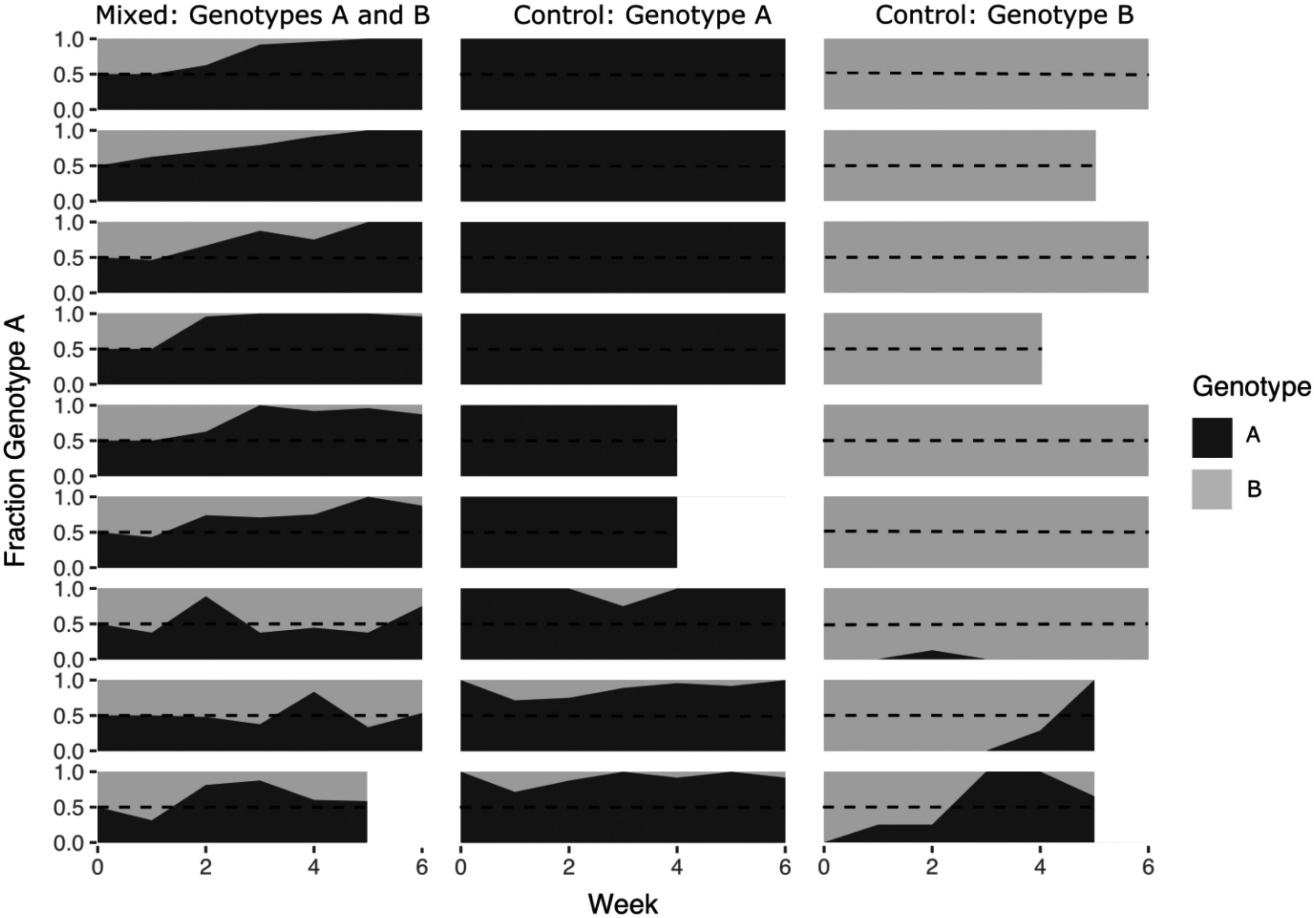
Changes in genotype frequency in mesocosms over 6 weeks, where genotype A is in black and genotype B is in gray. Dashed lines represent equal amounts of each genotype. Mesocosms in which populations went extinct before the end of the experiment are left blank after extinction.

Genotype A increased in frequency in the mesocosms with a mixture of the two genotypes (Figure 1). In the final week of the experiment, the fraction of genotype A was significantly greater than the starting fraction of 0.5 according to the Wilcox signed‐rank test (*p* = .014). By that time, genotype A had reached fixation in 3 of the 8 remaining populations and was more abundant than genotype B in all populations.

**FIGURE 2:**
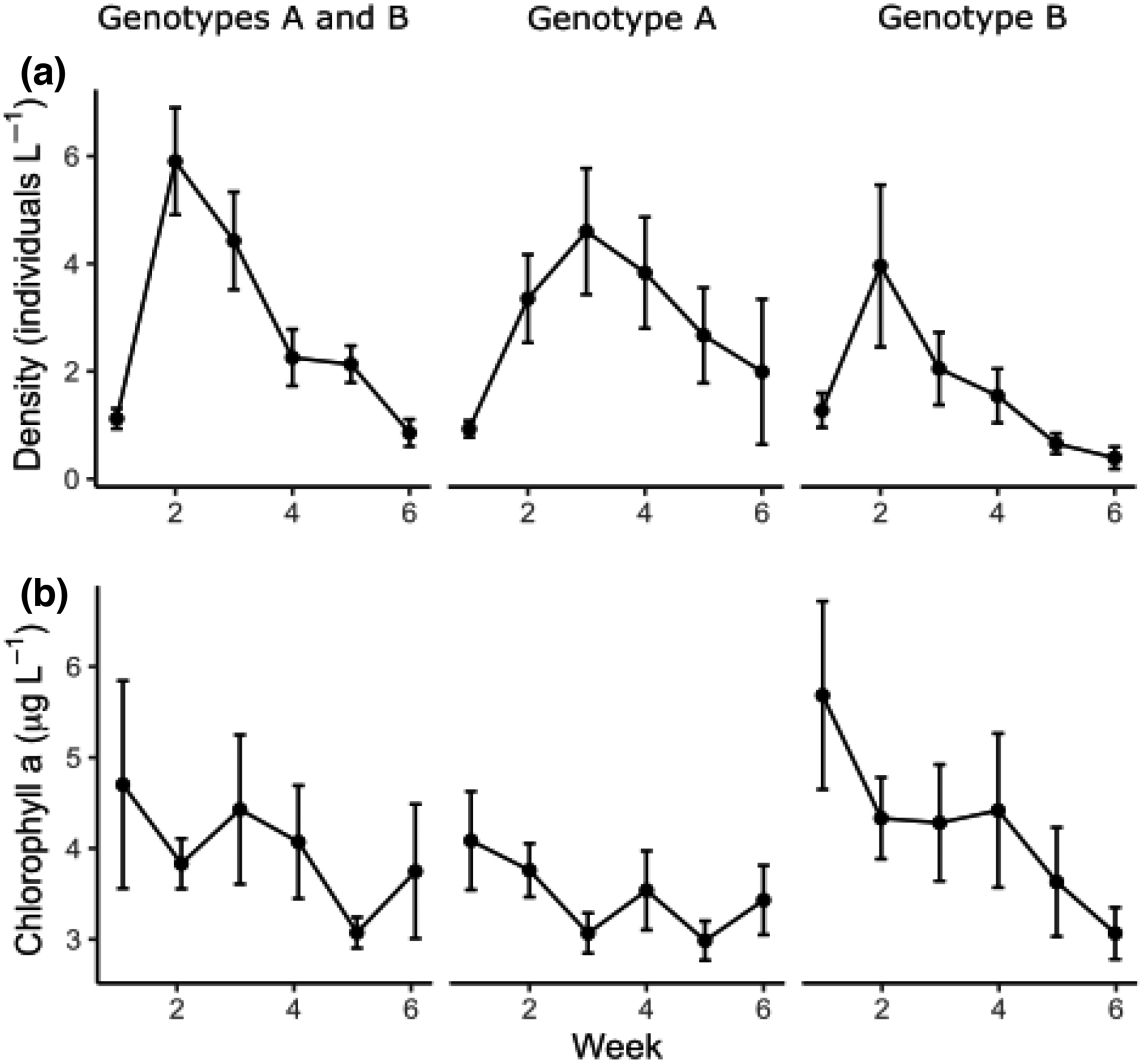
Mean *Daphnia* density (a) and chlorophyll a (b) ±1 SE in mesocosms over time by treatment.

Both genotypes were able to increase in density in the mesocosms when cultured individually (Figure 2a), with the exception of two mesocosms containing genotype A alone. Compared to the initial density of 1.56 L^−1^, populations with genotype A alone averaged a density of 2.7 L^−1^ (*SD* = 0.36), populations with genotype B alone averaged a density of 1.6 L^−1^ (SD = 0.28), and mixed populations averaged a density of 2.6 L^−1^ (SD = 0.30). Chlorophyll a in the mesocosms averaged 3.8 μg L^−1^ (SD = 1.73) and we saw a decline in chlorophyll a overtime, especially in the mesocosms with genotype B alone (Figure 2b).

There was evidence that some mesocosms that should have contained only a single genotype became contaminated (Figure 1). This accidental test of invasion when rare occurred in six mesocosms in total. Although we rinsed our sampling gear with zooplankton‐free water between each sample, the *Daphnia* may have been transferred between mesocosms in the process of sampling. Interestingly, in mesocosms in which a small number of individuals of genotype A were introduced, they were able to increase in frequency in two of three of these populations, whereas in the mesocosms in which individuals of genotype B were introduced, they declined to make up only 8% of the population or went extinct entirely. The inability of genotype B to invade when rare is consistent with the results of the mixed mesocosms in which genotype B declined or was excluded entirely.

**FIGURE 3:**
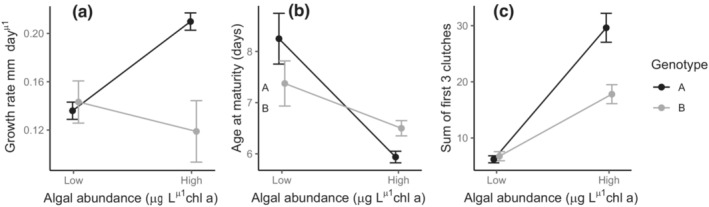
Plot of reaction norms across high‐ and low‐resource values for (a) growth rate, (b) age at maturity, and (c) reproduction measured as the sum of neonates produced in the first three clutches. Points are means with error bars corresponding to ±1 SE. Genotype A is in black and genotype B is in gray.

The life‐history assays showed that resource exploitation differed by genotype. Genotype A was more fit in the higher‐resource treatment compared to genotype B, in that it had a higher growth rate and produced more offspring (Figure 3, Table 2). In the low‐resource treatment, genotype B had an earlier age at maturity than genotype A (Figure 3, Table 2). The MANOVA showed there was a highly significant interaction between genotype and resources (*F*_1,32_ = 8.05, df = 3, *p* = .0004), and a significant interaction between genotype and resources for all three traits individually (Table 2). The MANOVA also showed the effect of genotype was significant (*F*_1,32_ = 3.26, df = 3, *p* = .0035), and the effect of treatment was highly significant (*F*_1,32_ = 41.39, df = 3, *p* < .0001). More specifically, on average, genotype A had a higher growth rate and more offspring overall compared to genotype B (Figure 3a,c). There was a non‐significant main effect of genotype on the age at maturity (Figure 3b). Also, the high‐resource environment reduced the age at maturity and increased reproductive output, although it did not have a significant effect on growth rate (Figure 3).

**TABLE 2:** Results of the individual ANOVAs, testing the effects of genotype, resource condition and their interaction on growth, ln‐transformed age at maturity, and ln‐transformed reproduction. Also shown are the pairwise comparisons between genotypes in high‐ and low‐resource treatments for the same three traits.

		Growth rate		Age at maturity		Reproduction	
	df	*F*	*p*‐Value	*F*	*p*‐Value	*F*	*p*‐Value
Genotype	1	6.01	**.020**	0.19	.66	4.44	**.043**
Resource	1	2.10	.16	20.61	**<.0001**	151.84	**<.0001**
Genotype × Resource	1	8.27	**.007**	5.92	**.020**	8.19	**.007**

Pairwise comparisons				
Trait	Resource	Mean difference between genotypes	SE	Adj *p*‐value
Growth rate	High	0.091	0.024	**.0006**
Growth rate	Low	−0.007	0.024	.77
Age at maturity	High	−0.090	0.065	.18
Age at maturity	Low	0.13	0.063	**.046**
Reproduction	High	0.52	0.15	**.002**
Reproduction	Low	−0.079	0.15	.59

## 4 DISCUSSION

We found evidence of selection favoring one genotype over the other. The same genotype increased in frequency across all experimental mesocosms (Figure 1). Especially, given that the modest population sizes we used could have been impacted by demographic stochasticity or genetic drift, the consistent trend across mesocosms suggests that changes in genotype frequencies are predictable, at least in the short term during the summer environmental conditions in this pond. Our results shed light on previous studies that have documented seasonal turnover in *Daphnia* genotypes in the wild (Carvalho & Crisp, 1987; Pfrender & Lynch, 2000; Steiner & Nowicki, 2019), in that some of this turnover is likely the result of selection favoring certain genotypes and not entirely driven by chance. It is worth noting that most natural *Daphnia* populations are genotypically diverse, including those in the studies above, and that we were only able to examine turnover in two dominant genotypes. Despite this, we established that, in this pond, turnover had a deterministic aspect, which made investigating the underlying drivers worthwhile.

We also found evidence for one possible process driving selection: an r versus K selection trade‐off among the two dominant genotypes in the pond. In previous studies, this has also been called a “power‐efficiency” trade‐off, that is, one genotype has “power,” rendering it better at exploiting abundant resources, whereas the other genotype has “efficiency,” rendering it better at exploiting scarce resources (Crawford et al., 2020; Raubenheimer & Simpson, 1996). We see some evidence of crossing reaction norms across the two resource conditions (Figure 3). Specifically, genotype A had a higher growth rate and more offspring compared to genotype B under high‐resource conditions, whereas genotype B responded slightly better to low‐resource conditions compared to genotype A. Although chlorophyll a declined in the mesocosms, resulting in lower‐resource conditions at the end of the experiment, there was no evidence that the direction of selection changed to favor genotype B. Given that genotype B was only significantly better with respect to one trait, development time, this might not be enough of an advantage to allow it to persist in the pond from which it was isolated, even under fluctuating environmental conditions. It is possible that this genotype only recently arose in early spring when dormant sexually produced eggs hatch and contribute new genetic variation into the population. Alternatively, there may be additional trade‐offs in traits we did not measure, such as the lifespan–reproduction trade‐off found among genotypes of *Daphnia magna* (Pietrzak et al., 2010). While chlorophyll a content in the pond during the experiment was likely intermediate compared to the low and high conditions in the life‐history assays, we can hypothesize from the mesocosm results that June–August months reflected environmental conditions in which resources are high enough that genotype A has higher fitness.

Our study is limited in the conclusions we can draw regarding how performance in the assays relates to performance in the pond mesocosms. Laboratory phytoplankton monocultures typically have higher fluorescence and higher nutritional value than the natural phytoplankton community, in which not all taxa are edible. Furthermore, our methods do not allow us to distinguish whether the changes in measured chlorophyll a in the field across mesocosms and over time were due to reduction in phytoplankton biomass or changes in other factors that affect fluorescence such as taxonomic composition, light intensity, and turbidity. Finally, we recognize our study is limited in scope to the two dominant genotypes in a single pond. Future research on the role of resource‐based trade‐offs in driving selection in additional populations and genotypes would be desirable. Nonetheless, our study provides a useful example of parallel evolution under natural conditions and points to a potential mechanism driving the repeatable outcome of selection observed in our field experiment.

Crossing reaction norms have been previously observed across species of *Daphnia*, in which species differ in their sensitivity to a resource gradient (Tessier et al., 2000). This trade‐off can be explained by differences in acquisition, in that certain species or genotypes, termed “superfleas,” are better at acquiring nutrients but only when resources are abundant (Hall et al., 2012; Reznick et al., 2000; Spitze et al., 1991). It has been termed a “power‐efficiency” trade‐off when describing resource acquisition strategies in rich‐ and poor‐quality environments or an “r‐ versus K‐selection” trade‐off when describing suites of life histories more generally. Given the similar pattern seen in our results, we believe the same mechanisms operating at the species level are also operating at the genotype level. Genotype‐specific differences in response to food availability (Glazier, 1992; Hall et al., 2012; Pietrzak et al., 2010; Plaistow & Collin, 2014) and food quality (Jeyasingh et al., 2009) have been previously documented in *Daphnia* and other organisms (Osier & Lindroth, 2006; Turner et al., 1996). Our findings can be compared to the laboratory study of Weider et al. (2005), which not only found a genotype‐by‐environment trade‐off in resource use of two *Daphnia* clones but also parallel evolution of one genotype overtaking the other depending on whether nutrient conditions were high or low. On the other hand, our findings contrast with those of Crawford et al. (2020), which failed to show a power‐efficiency trade‐off between *Daphnia* clones in persisting versus spring‐only populations, or did they show a trade‐off between spring and summer clones within the same population.

An important implication of a resource‐based trade‐off is its potential to maintain coexistence among clones and hence, genetic variation within a population. Theory suggests coexistence is possible under a resource‐based trade‐off and one of two additional conditions. The first condition is if the functional responses to a resource gradient are curved, both genotypes can persist on a single resource (Armstrong & McGehee, 1980, see their figure 2). In the case of non‐linear saturating functional response curves, each genotype has an advantage when rare. If a small number of the low‐resource‐favored genotype enters a population of the high‐resource genotype at equilibrium, they can increase because the resource level is higher than what they require for positive growth, similar to the R* rule. If a small number of the high‐resource‐favored genotype enters a population of the low‐resource genotype which undergoes internally generated cycles, they can increase during periods of high resource levels.

The second condition is if genotype‐by‐environment interactions are paired with temporally fluctuating resources, for example, driven by seasonality (Miller & Klausmeier, 2017), or spatial variability in resources (Amarasekare, 2003) would also allow for coexistence through fluctuating selection (Abrams, 2022; Gillespie & Turelli, 1989; Haldane & Jayakar, 1963; Lynch, 1987; Schreiber, 2020). Evidence from our mesocosms suggests genotype B is not able to invade when rare in the summer and this would prevent coexistence if environmental conditions remained constant. Yet, ponds are dynamically changing environments. Therefore, multiple outcomes are possible: either genotype B is not completely excluded and the environment changes to favor it, allowing coexistence, or genotype B goes extinct and genotypic variation must be renewed yearly from sexually produced eggs hatching. In the *Daphnia longispina* complex, it has been found that parental types and their hybrids, which differ in life history, are able to coexist due to temporally changing environments (Spaak & Hoekstra, 1995, 1997). However, *D. longispina* clones typically do not persist for multiple years (Yin et al., 2010), so genetic variation seems to be maintained by a combination of coexistence and new input. Dynamics may differ in the pond studied here, as it does not freeze in winter and *D. pulex* are present year‐round, which may promote the persistence of multiple clones.

As resources in aquatic environments exhibit predictable seasonal patterns of algal succession (Sommer et al., 1986), there is the potential for seasonal resource partitioning (Schoener, 1974). Carvalho and Crisp (1987) demonstrated that five of the dominant genotypes in their system were seasonal specialists, favored in summer, fall, or winter, and that this pattern was consistent across multiple locations within a lake and across multiple years. In ostracods, this seasonal resource partitioning is thought to allow for coexistence among lineages (Rossi et al., 2017). There are a vast number of seasonally changing variables that could be responsible for the seasonal turnover of genotypes, with resource availability being just one possibility. For example, previous studies have documented genotype‐specific responses to environmental variables such as temperature, phosphorus limitation, and salinity (Carvalho, 1987; Sherman et al., 2017; Van Doorslaer et al., 2009; Venâncio et al., 2018). One implication of this pattern is that we may expect climate change to induce changes in genotype frequencies. Frequencies could be affected by both seasonal and spatial resource partitioning associated with warming and changes in productivity or the phenology of productivity. Clones favored in winter may be permanently replaced by those favored in summer. Biotic factors such as predation and parasitism, although not present in our mesocosms, may also play a role in maintaining genotypic variation in natural populations of *Daphnia* (Hall et al., 2007; Tseng & O'Connor, 2015; Walsh & Post, 2012) and other organisms (e.g., Jokela et al., 2003). Future studies should explore how these other variables not only drive selection but also how they interact with, or mediate, resource selection.

Overall, we found that evolution was parallel across mesocosms and that resource exploitation differed by genotype. The genotype favored in the mesocosms was also the genotype that performed better at high resources. These findings are consistent with observational studies of seasonal turnover, in which specific genotypes are favored during specific seasons. They are also consistent with experimental studies of resource‐based trade‐offs, in which genotypes differ in life‐history strategies. This study adds to the literature by documenting parallel evolution in a natural setting and is unique in that mesocosm populations are initiated with identical genetic material on which selection can act. It also begins to inspect resource availability as a driving force of the observed evolution. We hope to inspire further studies that combine field experiments and lab assays as they will be especially powerful in developing a more complete understanding of the repeatability of evolution, fluctuating environmental conditions, and their potential to promote coexistence.

## AUTHOR CONTRIBUTIONS

**Kelsey Lyberger:** Conceptualization (lead); data curation (lead); formal analysis (lead); writing – original draft (lead). **Thomas Schoener:** Supervision (lead); writing – original draft (supporting); writing – review and editing (equal).

## CONFLICT OF INTEREST STATEMENT

The authors declare no competing interests.

## Data Availability

The data that support the findings of this study are openly available in Dryad at https://doi.org/10.25338/B8J340.

## Appendix

**TABLE A1:** Temperature and composition of zooplankton in the pond. Temperature was recorded just below the surface and at 3 m depth. We failed to record temperature in week 5. Two vertical zooplankton tows were taken from the center of the lake in May 2018 and adjacent to the mesocosms at all other time points. Major taxonomic groups of mesozooplankton were counted using a stereomicroscope at 20× magnification and reported as number of individuals per liter. Rare taxa and microzooplankton were ignored.

Week	Temperature (°C) 3 m	Temperature (°C) surface	*Daphnia pulex*, L^−1^	Calanoid copepods, L^−1^	Cyclopoid copepods, L^−1^	Harpacticoid copepods, L^−1^	*Simocephalus* spp., L^−1^
May 2018	13	17.5	8	1.8	0.09	0.27	0.22
0	16.1	21.4	2.88	1.51	0.05	0.13	0.06
1	16.2	22.1	0.22	2.24	0.26	0.48	0.16
2	16.7	23	1.78	1.88	1.5	0.86	0.02
3	17.1	24.5	4.52	4.48	1.02	0.36	0.12
4	18.9	24.8	7.3	0.78	2.12	0.7	0.01
5	NA	NA	2.8	0.72	0.76	1.16	0.04
6	21.5	26	0.74	0.8	0.2	0.38	0.02

**TABLE A2:** The probability of two individuals having identical genotypes at each loci and across all 5 loci. Allele frequencies are given for the 16 genotyped individuals. Probabilities are calculated using methods in Taberlet and Luikart (1999).

Locus	Allele	Frequency	Probability of identity
CAA8	130	0.3125	.07
CAA8	136	0.1875	
CAA8	142	0.3125	
CAA8	148	0.1875	
GTT3	198	0.28125	.35
GTT3	202	0.71875	
CAA27	199	0.1875	.06
CAA27	200	0.03125	
CAA27	203	0.28125	
CAA27	205	0.1875	
CAA27	206	0.3125	
CAA2	213	0.21875	.13
CAA2	223	0.3125	
CAA2	224	0.46875	
CAA14	240	0.3125	.15
CAA14	243	0.5	
CAA14	246	0.1875	
Multilocus probability of identity			3.01 × 10^−5^
